# IUC26665-90 Global inequalities in prostate cancer incidence and mortality: an ecological analysis

**DOI:** 10.1093/oncolo/oyag286.013

**Published:** 2026-08-21

**Authors:** M Hosseini, Y Vaez-Gharamaleki, A Motamedi

**Affiliations:** Hematology and Oncology Research Center, Tabriz University of Medical Sciences, Tabriz - Iran, Islamic Republic of; Hematology and Oncology Research Center, Tabriz University of Medical Sciences, Tabriz - Iran, Islamic Republic of; Hematology and Oncology Research Center, Tabriz University of Medical Sciences, Tabriz - Iran, Islamic Republic of

## Abstract

**Background:**

To evaluate global inequalities in prostate cancer incidence and mortality across countries with different levels of human development.

**Methods:**

A cross-sectional ecological study was conducted using country-level data from the World Health Organization Global Cancer Observatory (v1.1) in June 2026. Age-standardized incidence rates (ASIRs) and age-standardized mortality rates (ASMRs) per 100,000 population in 2022 were extracted for prostate cancer. Human Development Index (HDI) was obtained from the United Nations Development Programme report 2022 for all available countries and used as a comparative macroeconomic indicator of socioeconomic development. Countries were classified into four categories of very high, high, medium, and low HDI. Mortality-to-incidence ratios (MIRs) were calculated for all countries, and global inequalities were assessed by comparing age-standardized rates and MIRs across HDI groups using Spearman's rank correlation coefficient. Statistical significance was defined as *P value* <0.05.

**Results:**

A total of 1,467,854 (95%UI: 1,457,501–1,478,281) new cases and 397,430 (95%UI: 391,946–402,991) deaths were estimated for prostate cancer in 2022 worldwide, with an ASIR and ASMR of 29.4 and 7.3 per 100,000 population, respectively. Countries with very high HDI demonstrated the highest ASIR with 57.7, whereas the highest ASMR (14) was observed in the low HDI group. On the country-level analysis, Lithuania, Norway, and Sweden had the highest ASIRs with 135, 109.9, and 104.3, whereas the highest ASMRs were observed in Chad, Zambia, and Zimbabwe, with 49.4, 37.6, and 37.2 per 100,000 population, respectively. Correlation analysis demonstrated a moderate positive association between HDI and the ASIR (ρ  =  0.456, *P* <0.001), a weak negative association between HDI and ASMR (ρ  =  -0.203, *P* <0.01), and a strong negative association between HDI and MIR (ρ  =  -0.838, *P* <0.001), indicating that higher HDI is strongly associated with lower MIR (Figure 1).

**Conclusions:**

Substantial inequalities in prostate cancer burden exist across the global development spectrum. Although countries with higher HDI experience greater prostate cancer incidence, lower HDI countries bear a disproportionately higher mortality burden and poorer outcome indicators.

**IUC26665-90 Figure 1:**
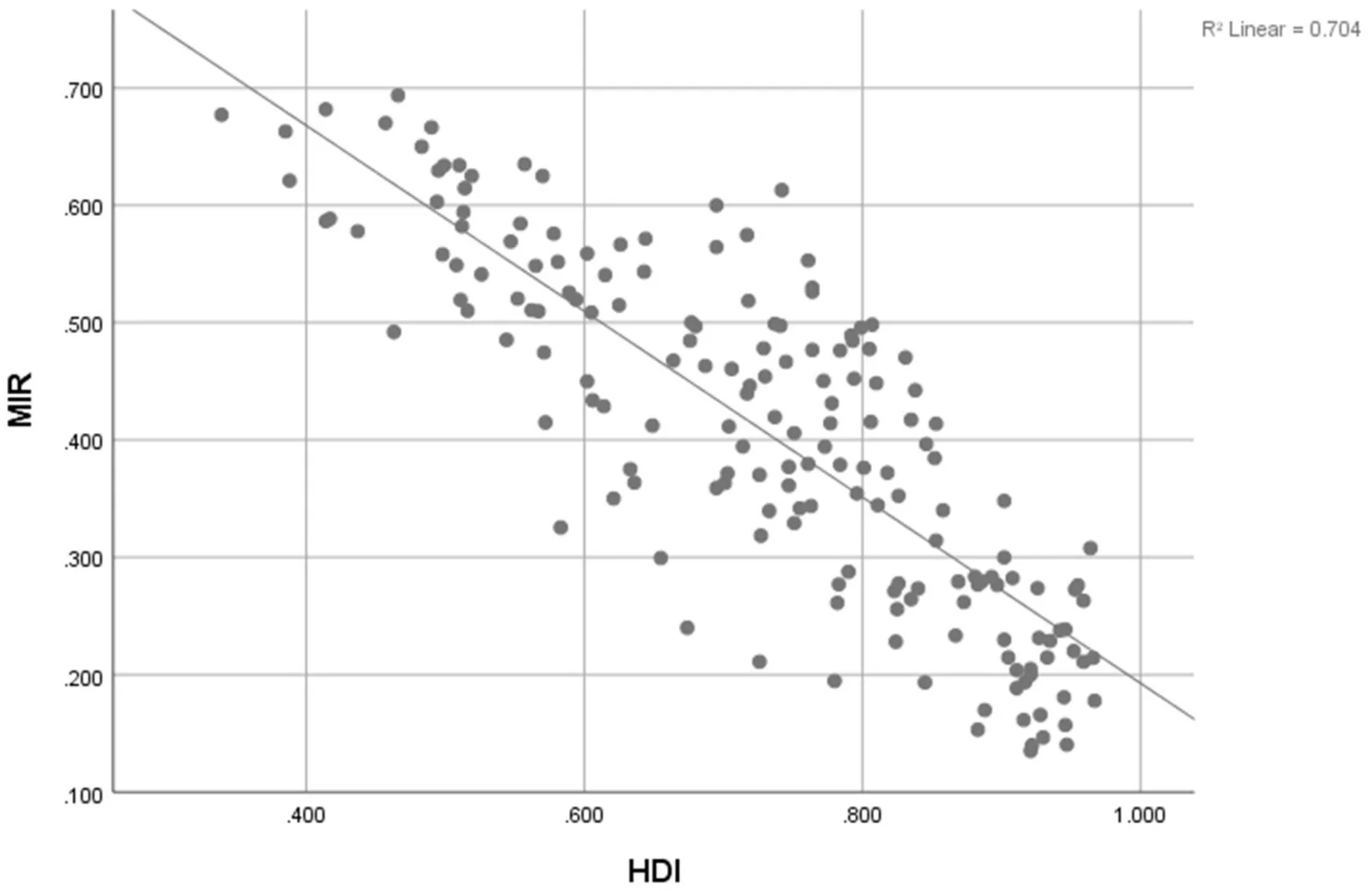
Scatter plot of the association between HDI and MIR for prostate cancer worldwide. The fitted regression line demonstrates the overall relationship between HDI and MIR.

